# Impact of Advanced Impregnation Technologies on the Bioactivity, Bioaccessibility, and Quality of a Hydrolyzed Collagen-Enriched Apple Snack

**DOI:** 10.3390/foods14050817

**Published:** 2025-02-27

**Authors:** Helena Nuñez, Rodrigo Retamal, Aldonza Jaques, Marlene Pinto, Pedro Valencia, Mónika Valdenegro, Cristian Ramirez, Sergio Almonacid, Andrés Córdova

**Affiliations:** 1 Departamento de Ingeniería Química y Ambiental, Universidad Técnica Federico Santa María, P.O. Box 110-V, Valparaíso 2390123, Chile; helena.nunez@usm.cl (H.N.); rodrigo.retamal@sansano.usm.cl (R.R.); aldonza.jaques@usm.cl (A.J.); marlene.pinto@usm.cl (M.P.); pedro.valencia@usm.cl (P.V.); cristian.ramirez@usm.cl (C.R.); sergio.almonacid@usm.cl (S.A.); 2 Programa de Doctorado de Ciencias Agroalimentarias, Facultad de Ciencias Agronómicas y de los Alimentos, Pontifica Universidad Católica de Valparaíso, Valparaíso 2340000, Chile; monika.valdenegro@pucv.cl; 3 Escuela de Agronomía, Pontificia Universidad Católica de Valparaíso, Calle San Francisco S/N, La Palma, Quillota 2260000, Chile; 4 Escuela de Alimentos, Pontificia Universidad Católica de Valparaíso, Waddington 716 Playa Ancha, Valparaíso 2340025, Chile

**Keywords:** hydrolyzed collagen, impregnation, bioaccessibility, refractance window

## Abstract

The increasing demand for functional foods with added health benefits has driven the development of innovative food products. This study aimed to develop a functional snack made from Granny Smith apples enriched with hydrolyzed collagen using impregnation technologies, including vacuum impregnation (VI), ultrasound (US), and moderate electric field (MEF), and pretreatment with CO_2_ laser microperforations (MPs) combined with drying methods, including conventional drying (CD) and refractance window drying (RW). The collagen content increased significantly across treatments, with MP-I achieving the highest retention (79.86 g/100 g db). Compared with VI-CD (3.8 mg GAE/g db), MP-RW drying resulted in more total polyphenols (up to 7.2 mg GAE/g db), which was attributed to its shorter drying time (55 min vs. 160 min). The RW treatments also better-preserved color quality, with higher a* (red tones) and b* (yellow tones) values, especially in the MP-RW and US-RW treatments, highlighting their advantages in maintaining visual appeal. Texture analysis revealed that RW drying produced slices with reduced hardness and increased crispness, with MP-RW resulting in the highest sensory crispness score (8.3). In vitro digestion demonstrated that the (VI) treatment resulted in the highest degree of collagen bioaccessibility (~90%), underscoring the effectiveness of this method in improving nutrient delivery compared with the 65% MP, ~70% US, and ~74% methods. The ~90% bioaccessibility is particularly noteworthy, as it indicates that a significant portion of the impregnated collagen remains available for absorption, reinforcing the potential of VI as a strategy for developing functional foods with enhanced nutritional benefits.

## 1. Introduction

New dietary trends worldwide reflect growing consumer interest in foods with high nutritional value and positively influence the physiological functions of the human body [1]. Driven by increasing concerns about health and quality of life, the demand for functional foods has increased, prompting the food industry to intensify its research into developing and applying these innovative products [2]. As one of the most valuable ingredients in the food industry, hydrolyzed collagen stands out for its versatility, exceptional nutritional properties, and proven benefits for skin health [3], joint support, alleviation of cardiovascular disease [4,5], and overall well-being. Collagen, the most abundant structural protein in animals and humans, comprises approximately 30% of the total protein content. Collagen synthesis naturally decreases with age, leading to an increased demand for supporting the health of skin, hair, and bone tissues [6]. Hydrolyzed collagen produced through acid, alkaline, or enzymatic hydrolysis of collagen or gelatin [7,8] has a lower molecular weight, making it more easily absorbed and potentially facilitating cartilage regeneration by supplying material for in situ collagen synthesis [9]. Some human studies and clinical research suggest that daily doses of 10–40 mg of undenatured collagen may improve joint health and reduce pain in individuals with hip or knee osteoarthritis [4,10,11,12]. Collagen has been utilized in various applications, including as a protein dietary supplement, a functional ingredient in meat processing [13], a component of edible films [14], fruit juice drinks [15], coatings for food products [16], and a food additive to increase product quality [17]. Currently, studies on the application of collagen impregnation in fruit-based products, particularly those involving the use of advanced impregnation techniques, are lacking. Although vacuum impregnation and other mass transfer methods have been explored for incorporating bioactive compounds into fruits, collagen has not been extensively studied in this context. The lack of literature on this topic underscores the need for further research to explore the interaction of collagen with fruit matrices, its stability during processing, and its potential to enhance the functional and sensory properties of fruit-based products. In this context, plant tissues, particularly fruits, offer a promising platform for developing fortified snacks because of their natural structure and rich nutrient composition [18,19]. However, integrating nutrients into cell-structured foods poses a significant challenge.

Several innovative technologies have been investigated to improve the incorporation of bioactive compounds into plant matrices. These technologies modify the structural properties of plant tissues, increasing permeability, mass transfer, and the retention of nutrients or bioactive components within the matrix. For example, vacuum impregnation (VI) creates a pressure differential that removes air and interstitial fluids from plant tissue pores, replacing them with an impregnating solution. Its impact on cell integrity is minimal, as it primarily facilitates passive diffusion into intercellular spaces without significantly altering cell walls [20].However, a complementary technique to increase tissue permeability and incorporate higher concentrations of the active compounds is usually needed. VI has been used to include blueberry juice in an apple matrix [20] and for the development of calcium-fortified potato chips with high sensory acceptability [21]. Ultrasound (US) induces cavitation, generating mechanical forces that disrupt cell walls and open intercellular spaces, facilitating solute penetration. It can also affect intracellular membranes, promoting more uniform solute distribution and enhancing bioactive compound absorption [22]. It has been demonstrated to enhance the impregnation of apple cubes with vitamin B12, achieving high vitamin loads (0.12–0.19 mg/g db) [23], and its use in combination with VI increased the iron content of potatoes by up to 210% [22]. CO_2_ laser microperforation (MP) creates microscopic openings on the tissue surface, reducing resistance to mass transfer by disrupting the epidermal and parenchyma layers [24] This enhances solute incorporation and retention while promoting faster dehydration during drying [25] without compromising product quality and improves the retention of bioactive compounds [24,25,26]. In addition, the combined use of CO_2_ laser microperforation and vacuum impregnation (VI) has been evaluated as an effective method for accelerating the marination process. However, despite their benefits, this combined methodology has been reported in only meat matrices such as pork [27], chicken marinade [28], and salmon salting [29], with no existing reports of its use for the incorporation of compounds into fruit matrices. On the other hand, a technology that has been widely studied for the impregnation of compounds in plant matrices is moderate electric field (MEF). MEF technology utilizes an alternating electric field, typically 0.1 to 1 kV/cm, to induce electroporation in cell membranes [30]. Electroporation occurs when the electric field creates pores in the cell membrane, increasing its permeability and facilitating the transfer of compounds [31]. MEFs can be either reversible or irreversible, depending on the strength of the electric field [32]. In summary, several technology alternatives can be explored by themselves or in combination to increase the incorporation of valuable bioactive compounds into vegetable matrices, as they act by different mechanisms.

Although consuming fresh fruits is ideal for maximizing health benefits, their limited shelf life has driven research into processing methods that ensure year-round availability. Drying provides numerous advantages for preserving enriched fruit snacks, such as reducing storage, packaging, and transportation costs while significantly extending shelf life. In this context, refractance window drying (RW) is an innovative dehydration technique widely used for preserving heat-sensitive foods, such as fruits, vegetables, and juices [33,34,35]. This method spreads the product as a thin layer on a transparent polymer film (e.g., Mylar) that floats over hot water. Drying occurs through conduction and radiation, as the film transfers thermal energy from hot water to the food material, with minimal exposure to high temperatures, making RW ideal for retaining nutrients, color, and flavor. Its energy efficiency, short drying time, and ability to produce high-quality powders and concentrates have made refractance window drying a promising alternative for developing functional and enriched food products.

Notwithstanding the stabilization processing technologies used, consumers expect the food they consume to have the best nutritional value. In this context, bioaccessibility refers to the fraction of a nutrient or bioactive compound present in a food that is released and available for absorption in the gastrointestinal tract during digestion. Bioaccessibility represents the amount of a substance that the body can potentially utilize after ingestion [36]; without sufficient bioaccessibility, the body cannot fully realize the health-promoting benefits of these compounds, including antioxidants, vitamins, or proteins such as collagen.

As a result of the above mechanisms, different compound impregnation pretreatments have distinct impacts on bioaccessibility, nutritional quality, and sensory properties, necessitating a detailed evaluation of their effects to optimize the final product. For example, VI increases collagen loading through pressure differentials, enhancing the transport of the solution into the apple matrix, whereas MP improves tissue permeability by creating microperforations, allowing for greater retention of bioactive compounds. In addition, US increases collagen diffusion and penetration through acoustic cavitation, and MEF facilitates collagen incorporation because of the electroporation phenomena occurring in apple tissue. On this basis, it is hypothesized that the incorporation of hydrolyzed collagen into an apple matrix using advanced impregnation technologies, combined with innovative drying methods, will increase both the collagen load in the final product and its bioaccessibility. Similarly, conventional drying (CD) and refractance window drying (RW) may exert differential effects on the preservation of bioactive compounds, texture, and sensory properties. Therefore, the objective of this research was to evaluate the impact of VI, MP, US, and MEF in combination with RW to maximize collagen bioaccessibility, the retention of bioactive compounds, and sensory quality as a strategy for the development of a functional snack made from Granny Smith apples enriched with hydrolyzed collagen.

## 2. Materials and Methods

### 2.1. Apple Snack Preparation

Granny Smith (*Malus domestica*) apples were purchased from a local market to develop snacks and stored at 2 °C until use. This variety was selected given its high uniformity and porosity, making it well suited for the impregnation process [37]. The samples were peeled with a conventional kitchen knife and ground with a stainless-steel grater, resulting in slices 0.004 ± 0.0003 m thick and 0.04 ± 0.0001 m in diameter. To avoid enzymatic browning, a blanching process was performed in which the grated apple was immersed for 1 min in distilled water at 90 °C, followed by immersion in cold distilled water for 1 min. The impregnation solution was prepared with hydrolyzed collagen (Peptinex B, Gelnex, Itá, Brazil) enzymatically derived from beef diluted in water (275 g hydrolyzed collagen/L solution) to obtain an apple/impregnation solution ratio of 1:5 (*w*/*v*) in all impregnation treatments. The resulting samples after the impregnation treatments with the different technologies, before being subjected to drying, were referenced as I, to differentiate them from the fresh apple samples. In this way, the resulting samples after impregnation under vacuum, with microperforation, ultrasound, and with moderate electrical pulse fields have been abbreviated as VI-I, MP-I, US-I, and MEF-I, respectively.

### 2.2. Impregnation

#### 2.2.1. Vacuum Impregnation (VI)

The apple slices were soaked in the impregnation solution for 15 min. Typically, in VI systems, the highest impregnation levels occur at the lowest pressures, typically ranging from 50 mBar to 400 mBar in the vacuum chamber [38]. Based on preliminary tests conducted in this study, a vacuum pressure of 20 kPa was selected to optimize the impregnation process while preserving the structural integrity of the apples. The process was performed using a DVP-1 vacuum pump (Dosivac, Buenos Aires, Argentina). The samples were then kept in the impregnation solution for 15 min, reestablishing atmospheric pressure. As this technology has been widely used for the incorporation of bioactive compounds, it can be referred to as a control treatment with respect to the impregnation stage.

#### 2.2.2. CO_2_ Laser Microperforation (MP)

The laser microperforations (MPs) were applied to the samples using a SYNRAD TI100 CO2 laser (Firestar t100, Synrad Inc., Mukilteo, WA, USA) with a 125 µm lens, which was linked to the computer with WinMarkPro Laser Making Software, allowing for configuration and adjustment. In accordance with [25], the operation conditions of the laser were set to achieve a pore diameter of 600 ± 15 μm (10 W, 120 pulses with a duration of 2 μs). The pore density (PD, the number of pores per unit area) configuration was conducted using 25 pores/cm2 in honeycomb arrangements. The pore size was measured using optical microscopy (H 600 LL HP 100, Hund Wetzlar, Wetzlar, Germany) with the uEye Cockpit software from Imaging Development Systems 113 (IDS) and Image-Pro Plus, which allows a photo of a microscopic observation to be captured [27]. Microperforation was used as a pretreatment for vacuum impregnation and was performed as described in Section 2.2.1.

#### 2.2.3. Ultrasound (US)

The apple slices were immersed in impregnation solution for 15 min, after which US treatment was applied using a Q700 ultrasonic generator (QSonica, Newtown, CT, USA) with a power output of 700 W and an ultrasonic fixed frequency of 20 kHz. The device was equipped with a 20 mm titanium probe, which was immersed 5 mm below the surface of the impregnation solution. The literature suggests that high-power ultrasound can produce severe cavitation effects on food structures at ultrasonic intensities above 10 W/cm^2^ and frequencies ranging from 20 to 100 kHz [39]. On this basis, the pulse duration was fixed at 1 s, and an amplitude setting of 57% was applied to achieve a power output of 400 W, thus producing an ultrasonic intensity of 127.4 W/cm^2^. The temperature of the impregnation medium increased during the treatment, and it was controlled with a temperature sensor immersed in the solution, reaching approximately 30 °C by the end of the sonication process. US was applied for 15 min, after which the samples were incubated for an additional 15 min to achieve a total immersion time of 30 min, as used for the other impregnation pretreatments [40].

#### 2.2.4. Moderated Electric Field (MEF)

The apple samples were immersed in impregnation solution in an MEF cell formed by two concentric cylindrical stainless-steel covers with graphite to prevent corrosion. The covers had diameters of 0.037 and 0.19 m and were fixed using a nonconducting plastic bottom. The distance (d) between the electrodes was 0.0765 m. The impregnated solution was subjected to an alternating current; the electrodes were connected using copper bridges to a 60 Hz voltage variator (Variac Slideup, Model SB-10, Niles, IL, USA) with a 220 V alternating current (AC) input. Furthermore, voltage and current transducers were used to measure the voltage and current through the samples. The temperature (30 ± 1 °C) and voltage were measured with a type T thermocouple connected to a data acquisition system (OM-320 data logger, Omega Engineering, Stamford, CT, USA). The temperature of the solution was held constant using a circulating refrigerated bath (MX20R-30-A12E, PolyScience, Niles, IL, USA). The voltage used in this research was determined in preliminary tests and was 100 V. MEF was applied for 15 min, followed by a 15 min period at atmospheric pressure.

### 2.3. Drying Process

#### 2.3.1. Conventional Drying (CD)

The apple slicing and drying process was performed in a convection oven (MEMMERT, model UFB400, Büchenbach, Germany) until the samples reached a moisture value of less than 10% [41]. The drying temperature was 65 ± 0.5 °C for 160 min. Drying was performed in triplicate. Given that this is a mature technology industrially used, it can also be referred to as the control process during the drying stage.

#### 2.3.2. Refractance Window (RW)

A thermoregulated bath (MEMMERT, model WNB22, Büchenbach, Germany) with distilled water at 65 ± 0.1 °C was used. A 0.1 mm thick plastic film (MYLAR, polyethylene terephthalate) was placed in the thermoregulated bath where the apple slices were placed. Drying experiments were performed in triplicate. Drying was performed until the samples reached a moisture content of less than 10% [41]. The drying time was 55 min.

### 2.4. Analytical Determinations: Water Activity and Moisture

Water activity (aw) was measured using a Rotronic brand hygrometer (HygroPalm HP23-AW-A, Bassersdorf, Switzerland) from 0.005 to 1.000 with an accuracy of ±0.005. The moisture content was determined according to AOAC method 934.06 [42] using an analytic balance (NANBEI, Model JD400-3, Zhengzhou, China) and a stove (MEMMERT, model UFB 400, Schwabach, Germany).

### 2.5. Color

Color measurements were performed using a colorimeter (Konica Minolta, model CR-400, Tokyo, Japan). The device records the parameters of the CieL*a*b* color space, where “L*” represents lightness, “a*” represents the red/green hue, and “b*” represents the yellow/blue hue. The color difference between a specific and reference sample is determined using Equation (1) [43], where $L_{0}^{*}$, $a_{0}^{*},$ and $b_{0}^{*}$ represent the color parameters of the reference. Δ*E* values indicate how perceptible the difference is to the human eye. Specifically, lower values indicate smaller differences, whereas higher values indicate greater differences. A ΔE higher than 5 indicates noticeable differences. The color difference (ΔE) of the impregnated apple was determined by comparing it with that of the fresh apple, whereas the dry samples were compared with the impregnated apple. This approach allows the assessment of color changes resulting from both impregnation and drying processes.

(1) $$∆E^{*}=\sqrt{{\left(L_{1}^{*}-L_{0}^{*}\right)}^{2}+{\left(a_{1}^{*}-a_{0}^{*}\right)}^{2}+{\left(b_{1}^{*}-b_{0}^{*}\right)}^{2}}=\sqrt{{∆L^{*}}^{2}+{∆a^{*}}^{2}+{∆b^{*}}^{2}}$$

### 2.6. Texture

The mechanical properties of the dried apple slices were evaluated according to [40] using a puncture test. Measurements were conducted with a Brookfield Texturometer (Model CT3, Middleboro, MA, USA) fitted with a 2 mm diameter cylindrical puncture probe (TA-39) and controlled using TexturePro CT software (version 1.6). Each apple slice was carefully placed and held on a special platform while the measuring tip penetrated the slice’s center at a constant speed of 2.0 mm/s with a trigger force of 0.1 N.

Sample thickness was measured in millimeters (mm). The maximum force was recorded in newtons (N), and crispness was determined based on the distance to reach the maximum force in millimeters (mm). Each treatment was tested ten times to ensure accuracy and repeatability.

### 2.7. Determination of Total Phenolic Content (TPC)

A spectrophotometric analysis was performed to determine the polyphenol content. A methanolic extract was prepared from 2 g of sample and 20 mL of an 80% methanol solution homogenized with a vortex mixer (FINEPCR, Modell FineVortex 2011, Gunpo, Republic of Korea) for 3 min. The extract was stored at room temperature and protected from light for 1 h, and the supernatant was filtered through Whatman paper MFS No. 231. The total phenolic content (TPC) of the extract was determined using the Folin-Ciocalteu assay following the methodology described by [44]. The absorbance was subsequently measured at 765 nm after incubation using a spectrophotometer (Spectronic Instrument, Inc., Model 336001, Rochester, NY, USA). A calibration curve was constructed using a gallic acid standard solution. The analyses were conducted in triplicate, and the results were expressed as mg gallic acid equivalents (GAEs) per gram of dry matter (db).

### 2.8. Determination of Proteins

The crude protein content of the samples was determined according to the AOAC 928.08 method [42].

### 2.9. In Vitro Bioavailability

GI digestion was performed according to the protocol developed by [45], sequentially simulating mouth, stomach, and small intestine digestion using a standardized method. In brief, in vitro digestion was conducted using a 5 g sample of dried apples. Salivary digestion was simulated using α-amylase solution and simulated salivary fluid (SSF). For the gastric digestion step (pH 3.0), pepsin and simulated gastric fluid (SGF) were added to the saliva, resulting in paste, and temperature-controlled shaking with agitation at 37 °C for 2 h was performed. Thereafter, the procedure continued with the simulation of intestinal digestion (pH 7.0) by adding pancreatin, bile salts, and simulated intestinal fluid (SIF), and the digestion mixture was stirred again for 2 h at 37 °C. After intestinal digestion, the samples were centrifuged for 10 min at 3500 rpm (Hermle Labortechnik Gmbh, Model Z323K, Wehingen, Germany), and the supernatants were collected for protein evaluation. The bioaccessibility percentage was used to analyze the effect of in vitro GI digestion on protein content. The bioaccessibility (% B) of the protein from each treatment of dried fruit was calculated using the following formula [46]:

(2) $$Bioaccessibility\left(\%\right)=(C_{digestion}/C_{indigested})\;\times 100$$

where C*_digestion_* is the concentration of the compound after G or GI digestion, and C_indigested_ is the concentration of the compound in the undigested sample.

### 2.10. Sensorial Analyses

Sensory evaluation was performed using a hedonic test methodology with 15 semitrained panelists between the ages of 21 and 25 years. The panelists assessed the samples’ color, crispness, firmness, acidity, aroma, sweetness, and general acceptability parameters using a scoring system ranging from 10 to 1 (10 points being extremely good and 1 being extremely poor). All procedures were developed according to the Guide for Sensory Analysis ISO 6658:2017 [47], considering the need for informed consent documents. To assess the repeatability and reproducibility of the panels, the standard deviation (SD) and the error standard (ES) were developed from the one-factor analysis of variance (ANOVA) of the panel means (grouped across sessions).

### 2.11. Statistical Analysis

The results were statistically analyzed using STATGRAPHICS Centurion XVI software, Statpoint Inc.^®^ (Warrenton, VA, USA), 2018. (ANOVA) and Duncan’s multiple range tests were performed to determine significant differences among the results with 95% confidence. The Levene test was also applied to assess homoscedasticity.

## 3. Results and Discussion

### 3.1. Collagen Content

**Figure 1:**
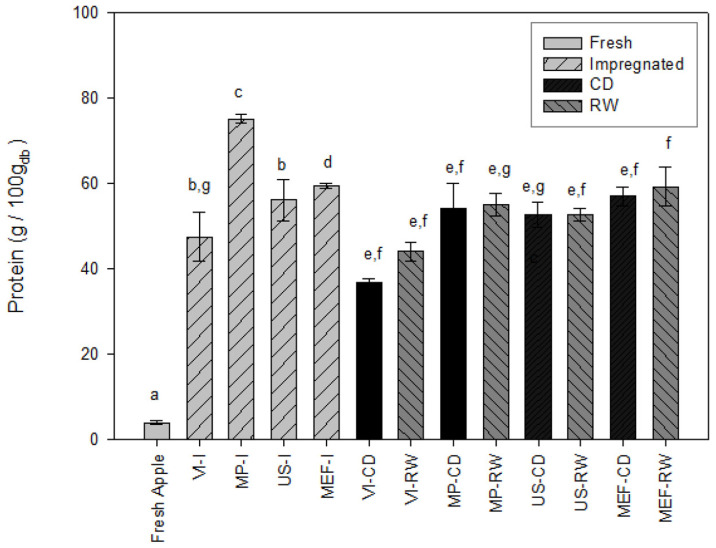
Effect of impregnation technology and drying process on protein content (VI: vacuum impregnation; MP: microperforation; US: ultrasound; MEF: moderated electric field; CD: conventional drying; RW: refractance window). Different lowercase letters indicate that the protein contents are significantly different (*p* < 0.05).

The protein content of fresh apples was 4.02 g/100 g dry base. Figure 1 shows the collagen content after the different treatments. As expected, all impregnation processes increased the protein content of the samples because of the exchange of the native liquid inside the pores with the collagen solution. VI-I, MP-I, US-I, and MEF-I significantly increased the protein content due to the addition of protein-rich impregnating solutions; the highest concentration was achieved with the MP-I treatment, reaching 79.86 g/100 g db. Vacuum impregnation (VI) was less effective than other technologies, such as microperforation (MP), ultrasound (US), and moderate electric field (MEF), in enhancing protein content impregnation in apple slices. This can be attributed to the limited structural modification caused by VI, which relies primarily on pressure differences to infuse the solution (collagen in this case) into the porous structure of the tissue [48]. Unlike MP, which creates small perforations, or US and MEF, which disrupt cell walls through cavitation or electroporation, VI does not significantly increase tissue permeability. As a result, the penetration and uniform distribution of the impregnating solution may be limited. The structural modifications introduced by MP, US, and MEF allow more profound and more effective diffusion of proteins into the tissue, resulting in higher retention during subsequent drying processes. These findings highlight the importance of selecting structural modification technologies to maximize the efficiency of impregnation and protein enrichment in processed apple slices. Among impregnated samples, treatments combined with RW drying resulted in greater protein retention compared with CD drying; however, there was no significant difference (*p* > 0.05).

### 3.2. Water Activity and Moisture Content

The moisture content and water activity of the collagen-impregnated apple samples are shown in Table 1. Compared with those of the fresh samples, both the moisture content and water activity decreased following all impregnation processes, indicating that dehydration occurred through diffusion between the impregnation solution and the water within the apple slices as the system reached osmotic equilibrium. This dehydration effect was likely enhanced by performing the impregnation process immediately after blanching. This resulted in greater destruction of the cell walls, reducing their structural integrity and consequently lowering their resistance to moisture loss [49]. The water activity and moisture content of the dried samples ranged from 0.325 to 0.368, and 0.036 to 0.080 g water/g samples effectively inhibited microbial growth [50]. The moisture content was significantly lower for the MP-I and MEF-I samples, as this pretreatment promoted a higher drying rate. The same impregnation method was applied across both drying technologies, and no significant differences (*p* > 0.05) in moisture content were observed. However, to achieve the final moisture content, the drying times for conventional drying were significantly longer than those for RW drying.

### 3.3. Color

**Figure 2:**
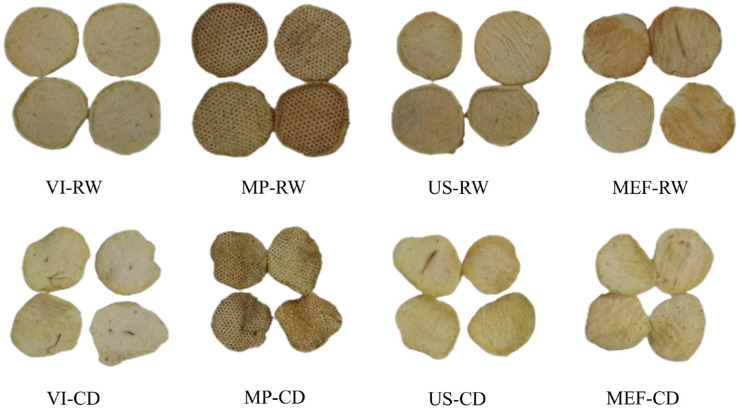
Pictures of apple slices after drying (VI: vacuum impregnation; MP: microperforation; US: ultrasound; MEF: moderated electric field; CD: conventional drying; RW: refractance window).

The color of a product influences its quality [49]. The effects of apple slice impregnation with hydrolyzed collagen on the color parameters L*, a*, and b* are presented in Table 2. The data presented in the table confirm that the impregnation processes and drying influenced the color change. In the case of the VI-I and MP-I samples, the difference in lightness L* was statistically significant (*p* < 0.05) compared with that of the fresh apples. VI-I significantly increased the lightness, indicating a lighter appearance than that of the fresh sample. These results contrast those obtained by other authors for apple impregnation with green tea extract [51] and aloe vera [52] as these studies reported darkening. The impregnation solution with the collagen hydrolysate could explain this phenomenon because of its transparency and light-reflecting properties. When introduced into a material’s porous structure, hydrolyzed collagen reduces light scattering by filling the spaces with a clear, reflective solution, which could increase the brightness of the material. In contrast, the CO_2_ microperforation treatment (56.89 ± 2.05) significantly decreased the L value, resulting in a darker appearance than that of the fresh sample because of the increased scattering of light caused by the uneven and disrupted surface created by the perforations. These microperforations alter the smooth texture of the slices, reducing the uniform reflection of light. For the MEF-I and US-I apple slices, no differences were observed between the impregnated and fresh samples (*p* ≥ 0.05). The comparison between CD drying and RW drying revealed significant differences (*p* <0.05) in their effects on the a* values for the MP, US, and MEF treatments. The CD treatments generally result in less pronounced shifts toward red tones than their RW counterparts do, potentially because CD involves longer drying times than RW does, which can increase browning reactions due to prolonged exposure to heat [53]. The b* value significantly differed among the treatments, reflecting changes in the yellow tones of the apple slices. VI slightly increased the yellow tone, with higher values observed after drying, particularly with RW, which preserved yellow pigments more effectively than did CD. Microperforation (MP) intensified the yellow tones significantly after drying, especially with RW (29.48 ± 0.42), suggesting increased pigment concentrations under gentler drying conditions. Ultrasound (US) treatments resulted in even higher b* values after drying, with RW (30.88 ± 0.52) yielding the most pronounced yellow tones because it preserves and releases carotenoids from disrupted cells. The moderate electric field (MEF) treatments resulted in similar trends, where RW drying (29.48 ± 2.49) consistently led to higher b* values compared with CD drying. These results indicate that RW drying is superior in retaining and intensifying yellow pigments across different pretreatments, likely because its milder conditions minimize degradation and browning reactions, enhancing the visual quality of the apple slices [54]. Similar to that noted for the a* value, the b* value for vacuum impregnation does not significantly differ between the drying treatments, which can be attributed to the uniformity of the VI process. VI effectively fills the porous structure of the apple slices with the impregnation solution, creating a relatively homogenous internal composition. This uniform distribution can minimize the impact of the drying method on the overall color (a* value) and other quality parameters, as the impregnated solution dominates the chemical and physical responses of the slices during drying; in contrast, treatments such as ultrasonication (US), microperforation (MP), and moderate electric field (MEF) introduce structural changes in the apple slices that are more sensitive to the drying method [22,55,56]. The total color change was calculated with Equation (1) using fresh apple slices as a reference, and the results are presented in Table 2. Pathare et al. [57] indicated that perceptible differences in color could be classified analytically for fresh and processed foods, considering ΔE > 3. This means that the consumer will have the impression of a completely different product color, which is also visible in the product photos (Figure 2). Changes in the total color difference (ΔE) parameter, with values that range from 6.53 to 18.48, were also observed. RW was slightly greater than that obtained for CD, but the difference was not significant (*p* ≥ 0.05), except for that for the samples treated with MEF. This phenomenon can be attributed to the structure induced by this treatment. MEF causes electroporation of cell membranes, which increases cell permeability and facilitates the release of intracellular compounds, including pigments such as carotenoids and polyphenols. This release makes the apple slices more susceptible to oxidation and browning reactions, particularly during drying [58].

### 3.4. Texture

The microstructure of fruits is strongly influenced by the drying technology, drying conditions, and final moisture content [45,59]. Table 2 summarizes the hardness, crispness, and thickness of collagen-impregnated apple slices dried using conventional drying (CD) and refractance window (RW) methods. Overall, conventional drying resulted in slices with significantly greater hardness, crispness, and thickness compared with the refractance window method, with notable differences (*p* < 0.05) observed for the US, MEF, and MP samples. Although conventional drying yielded the highest hardness for the VI samples, it yielded lower crispness. The textural differences between the two drying methods can be attributed to the impact of hot air drying, which typically causes substantial shrinkage and leads to the formation of a dense structure [60]. In contrast, the refractance window method, characterized by heterogeneous and rapid moisture diffusion, reduces shrinkage and thickness, resulting in lower hardness [61]. Additionally, the treatment method and the choice of impregnation solution influence the cellular tissue, leading to changes in its turgor and firmness [62]. In this study, VI, US, and MEF demonstrated similar textural characteristics. Moreover, the MP-I samples exhibited a rigid and crisp matrix, leading to reduced hardness but increased crispness. This phenomenon can be attributed to structural modifications caused by microperforation, which increase fragility, along with the slightly lower moisture content in the MP samples because of faster moisture diffusion during treatment [24]. This effect was more pronounced with conventional drying.

### 3.5. Total Phenolic Content (TPC)

**Figure 3:**
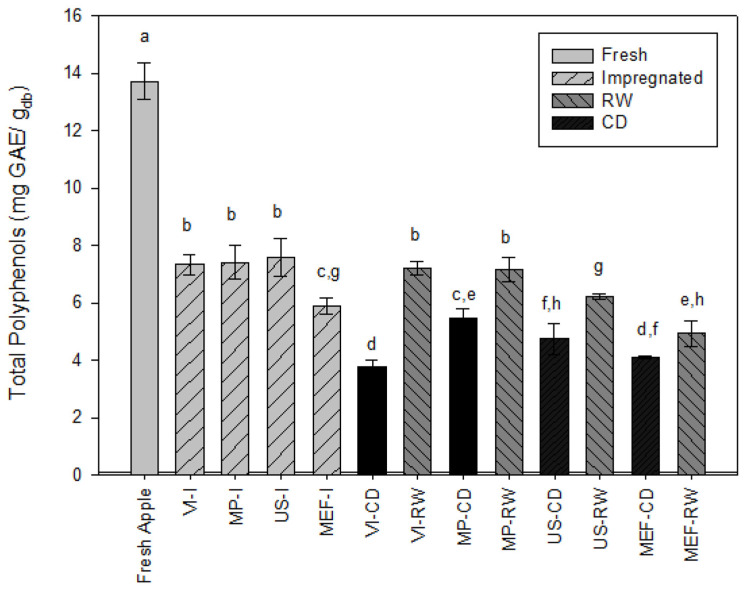
Effect of impregnation technology and drying process on total phenolic content (VI: vacuum impregnation; MP: microperforation; US: ultrasound; MEF: moderated electric field; CD: conventional drying; RW: refractance window). Different lowercase letters indicate that the total phenolic content is significantly different (*p* < 0.05).

Figure 3 shows the results of the total polyphenol content obtained from the treatments. The initial phenolic content (TPC) of the fresh apples was 13.70 ± 0.63 mg GAE/gdb, similar to that reported by [25] for Granny Smith apples. Compared with fresh apples, impregnated fresh samples (VI, MP, US, and MEF) contain fewer polyphenols. However, the VI, MP, and US treatments resulted in similar and relatively higher levels compared with the MEF treatment, leading to the lowest polyphenol content. The loss of polyphenols during impregnation could be due to their leaching into the impregnation solution [46]. Table 1 shows the decrease in moisture of the impregnated samples. Therefore, it may occur from two-way countercurrent mass transfer; the diffusion of water and other compounds, such as polyphenols from apple tissues into the impregnation solution; and the diffusion of collagen from the solution into the apple. Furthermore, VI, MP, and US are gentler processing methods, causing less degradation of polyphenols, whereas MEF likely introduces more significant thermal or mechanical impacts, resulting in greater losses; this could be explained by the fact that electroporation can increase the release of polyphenols, especially those bound to cell membranes [63]. This release also makes the polyphenols more susceptible to leaching in the impregnation solution. The drying process can significantly impact the retention of polyphenols, as observed in studies comparing different drying methods [64,65,66]. The comparison between CD and RW reveals significant differences (*p* < 0.05) in the total polyphenol content (TPC) of the apple slices. The TPC of apple slices dried with RW is greater than that of slices dried with CD, and this improvement can be attributed to the rapid heating and the significant reduction in drying time achieved by RW (55 min) compared with CD (160 min). A similar trend has been observed in studies comparing RW at different temperatures with the conventional drying of banana puree [67]. For apple slices, Rajoriya et al. [66] demonstrated that, compared with conventional methods, RW drying resulted in greater retention of phenolic compounds because of its ability to reduce the drying time and thermal degradation.

### 3.6. In Vitro Bioaccessibility

Determining the bioaccessibility of bioactive compounds is crucial for understanding their potential health benefits, as it indicates the fraction of these compounds that can be absorbed and utilized by the human body [68]. Table 3 shows the in vitro hydrolyzed collagen contents of the dried samples. The highest bioaccessibility values, close to 90%, were observed for VI-CD and VI-RW; these results are interesting because vacuum impregnation was the treatment with the least impact on collagen impregnation and the greatest bioaccessibility. The observation that higher impregnation processes result in lower bioavailability can be explained by interpreting in vitro digestion as a kinetic reactor process [69,70]. Although an increased substrate concentration enhances mass transfer due to steeper concentration gradients, the digestion process becomes less efficient at higher substrate concentrations when the digestion time is fixed (as per the protocol). As an additional factor, the duration of enzyme activity is limited. This inefficiency occurs because, at elevated substrate concentrations, the extraction process—releasing nutrients during in vitro digestion—is incomplete, thereby reducing overall bioavailability, measured as the percentage of nutrients extracted. With respect to the effect of the drying process, Table 3 shows no significant difference (*p* > 0.05) in bioaccessibility between the two drying methods, SC and RW, within the same processing treatment.

The type of drying process affects the release of bioactive compounds, and it is not possible to generalize whether one technology or another is better for the bioaccessibility of certain bioactive compounds. For example, the idea that the freeze-drying methodology is the best alternative as a conservation treatment for bioactive properties must be reviewed since studies have indicated that the convection-drying method works better in terms of the bioaccessibility of bioactive compounds such as total polyphenol content, total flavonoid content, and total anthocyanin content [71,72,73]. These results can be explained by the structural changes that occur in the matrix during the drying process. Information on bioaccessibility in emerging drying technologies remains limited. In the case of RW, greater retention of bioactive compounds has generally been reported; however, this is the first study determining the bioaccessibility of bioactive compounds (hydrolyzed collagen) obtained with RW drying technology.

### 3.7. Sensorial Analyses

**Figure 4:**
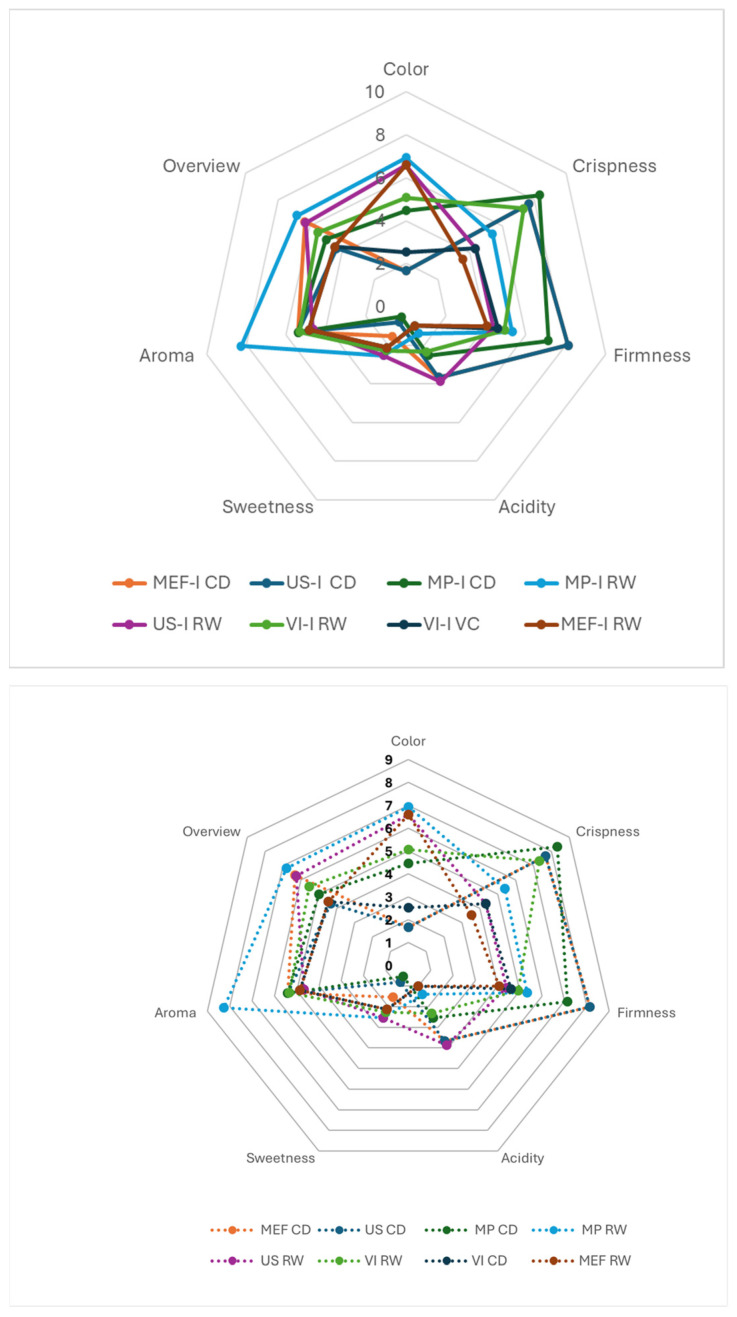
Sensory profile of impregnated dried apple samples.

In this study, collagen-enriched apple slices were produced as alternative healthy snack foods for human consumption. Therefore, collagen-enriched apple snacks should have acceptable sensorial quality properties. The sensory profiles of the samples, which are based on the mean values of each attribute evaluated by the panelists, are presented in Figure 4. The texture perception of the samples revealed that the CD samples presented greater hardness and crispness than the RW samples did, which aligns well with the results obtained from the texture analyzer. Similarly, the MP-CD sample, which presented the highest crispness in the texturometer, also presented the highest sensory crispness score of 8.3. In terms of hardness, the MEF-CD and US-CD samples were perceived as the hardest samples, with a sensory score of 8.1. However, the results of the texturometer indicated that the VI-CD samples had the highest hardness. Although all the samples had similar aroma scores, the MP-RW samples stood out significantly for having a more noticeable aroma than the other samples did, probably because of the burns induced by the laser on the samples during the microperforation process. In terms of color, consistent with the data obtained from the colorimeter, the RW samples were perceived to have a significantly more intense yellow color than the CD samples. Similarly, the MP-I RW and MP-I EST samples stood out because of their exceptionally pronounced color, which was likely attributed to the initial color change induced by the microperforation. In terms of flavor, the CD samples were perceived as slightly more acidic and less sweet than the RW samples. However, the overall scores were very low, as the panelists reported that the sweet/acidic flavors were masked by the collagen flavor, which was perceived as unpleasant. Finally, the overview of the samples indicated that the RW samples were slightly better rated than the CD samples were, with the MP-I RW sample receiving the highest score of 6.8 and the US-I EST sample the lowest score of 4.3.

## 4. Conclusions

The development of a functional snack made from Granny Smith apples enriched with hydrolyzed collagen demonstrated the effectiveness of combining advanced impregnation techniques (VI, MP, US, and MEF) with drying methods (conventional drying and refractance window drying). Although all the impregnation methods increased the protein content, the introduction of structural modifications (such as MEF and ultrasonication) facilitated better nutrient integration. Refractance window drying consistently outperformed conventional drying in preserving bioactive compounds, reducing drying times, and maintaining superior sensory properties. Despite the challenges of collagen flavor masking the natural sweetness of apple, the RW-dried, collagen-impregnated samples—particularly those pretreated with laser microperforation—achieved higher sensory scores, highlighting their potential as novel, healthy snack options. The in vitro digestion results revealed significant findings regarding bioaccessibility. Although vacuum impregnation had the least impact on collagen loading, it achieved the highest bioaccessibility (~90%). This outcome underscores the complex relationship between the substrate concentration, digestion efficiency, and enzyme activity, where excessive substrate concentrations may hinder complete nutrient release during digestion. This is the first study to determine the bioaccessibility of bioactive compounds, specifically collagen, using refractance window (RW) drying technology. Given the limited knowledge on this subject, further investigations are warranted to explore how processing techniques, such as RW drying, influence the structural characteristics of foods and, consequently, modulate the release and bioavailability of bioactive compounds. These findings provide valuable insights for optimizing food processing methods to increase the nutritional and functional qualities of food products.

## Figures and Tables

**Table 1:** Final moisture and water activity of fresh apple, impregnated apple, and impregnated dried apple samples.

Process	Moisture	Aw
Fresh sample	0.911 ± 0.001 ^a^	0.995 ± 0.003 ^a^
VI-I	0.869 ± 0.002 ^b^	0.935 ± 0.007 ^b^
VI-CD	0.070 ± 0.012 ^c,f^	0.362 ± 0.005 ^c^
VI-RW	0.069 ± 0.009 ^c,f^	0.330 ± 0.011 ^d^
MP-I	0.819 ± 0.020 ^d^	0.918 ± 0.002 ^e^
MP-CD	0.036 ± 0.003 ^e^	0.332 ± 0.009 ^d^
MP-RW	0.043 ± 0.020 ^e,g^	0.325 ± 0.015 ^d^
US-I	0.853 ± 0.009 ^b^	0.933 ± 0.005 ^b^
US-CD	0.065 ± 0.004 ^c,f^	0.361 ± 0.003 ^c^
US-RW	0.080 ± 0.008 ^f^	0.360 ± 0.005 ^c^
MEF-I MEF-SC	0.833 ± 0.012 ^d^ 0.056 ± 0.010 ^c,g^	0.924 ± 0.012 ^b,e^ 0.368 ± 0.004 ^c^
MEF-RW	0.074 ± 0.007 ^f^	0.360 ± 0.003 ^c^

Means with different superscripts within a column differ significantly (*p* < 0.05).

**Table 2:** CIELAB space parameters for color change in and textural properties of fresh apple, impregnated apple, and impregnated dried apple samples.

Process	L*	a*	b*	ΔE	Crispness (mm)	Thickness (mm)	Hardness (N)
Fresh sample	65.04 ± 1.88 ^a^	−5.25 ± 0.64 ^a^	14.60 ± 1.96 ^a^	-	-		
VI-I	71.07 ± 1.50 ^b,e^	−4.38 ± 0.57 ^a^	18.14 ± 1.76 ^b,f^	7.20 ± 2.50 ^a^	-		
VI-CD	75.83 ± 2.71 ^c^	−2.84 ± 0.79 ^b^	21.35 ± 0.5 ^c^	13.24 ± 2.10 ^b,c^	2.90 ± 1.11 ^a^	0.16 ± 0.02 ^a^	9.69 ± 1.54 ^a^
VI-RW	78.68 ± 3.12 ^c^	−2.90 ± 0.34 ^b^	22.68 ± 1.54 ^c^	16.10 ± 2.49 ^c,d^	2.28 ± 0.47 ^b^	0.1 ± 0.23 ^b^	3.56 ± 0.23 ^b^
MP-I	56.89 ± 2.05 ^d^	−2.71 ± 0.11 ^b^	20.95 ± 1.48 ^c,f^	10.86 ± 4.15 ^b^	-	-	-
MP-CD	55.23 ± 4.29 ^d^	−0.61 ± 0.74 ^b^	28.04 ± 1.25 ^d,e^	17.66 ± 0.54 ^d^	0.67 ± 0.11 ^c^	0.14 ± 0.01 ^a^	4.97 ± 0.74 ^c^
MP-RW	57.40 ± 0.95 ^d^	−2.42 ± 0.32 ^d^	29.48 ± 0.42 ^e^	18.48 ± 1.78 ^d^	1.9 ± 0.56 ^b,d^	0.11 ± 0.01 ^b^	3.47 ± 0.73 ^b^
US-I	66.60 ± 1.12 ^a,b^	−4.56 ± 0.78 ^a^	20.43 ± 1.34 ^b,c,f^	6.53 ± 0.43 ^a^	-	-	-
US-CD	70.48 ± 3.01 ^b^	−2.99 ± 0.38 ^b^	29.97 ± 0.88 ^e^	16.81 ± 1.44 ^d^	1.47 ± 0.78 ^d^	0.14 ± 0.02 ^a^	7.88 ± 1.01 ^d^
US-RW	75.29 ± 3.62 ^c,d^	−1.35 ± 0.41 ^c^	26.34 ± 2.94 ^d^	16.52 ± 1.97 ^c,d^	2.16 ± 0.64 ^b^	0.11 ± 0.01 ^b^	4.16 ± 0.6 ^ab^
MEF-I	68.21 ± 4.98 ^a,b^	−5.01 ± 0.69 ^a^	17.81 ± 2.45 ^b^	6.87 ± 1.45 ^a^	-	-	-
MEF-CD	68.71 ± 2.03 ^a,b^	−2.51 ± 1.07 ^b^	26.10 ± 2.04 ^d^	12.50 ± 0.49 ^b^	1.31 ± 0.68 ^d^	0.14 ± 0.02 ^a^	8.05 ± 1.73 ^d^
MEF-RW	70.47 ± 2.10 ^b^	−0.77 ± 0.76 ^c^	29.48 ± 2.49 ^e^	16.50 ± 0.84 ^c,d^	1.76 ± 0.46 ^b,d^	0.11 ± 0.01 ^b^	3.62 ± 0.61 ^b^

Means with different superscripts within a column differ significantly (*p* < 0.05).

**Table 3:** Bioaccessibility of impregnated dried apple samples.

Process	% Bioaccessibility
VI-CD	91.4 ± 0.92 ^a^
VI-RW	86.7 ± 0.17 ^a^
MP-CD	65.2 ± 2.48 ^b^
MP-RW	65.5 ± 2.48 ^b^
US-CD	70.4 ± 2.87 ^c^
US-RW	71.5 ± 0.65 ^c^
MEF-CD	74.3 ± 0.58 ^c^
MEF-RW	74.6 ± 3.46 ^c^

Means with different superscripts within a column differ significantly (*p* < 0.05).

## Data Availability

The original contributions presented in the study are included in the article, further inquiries can be directed to the corresponding author.
